# Aurantii fructus immaturus carbonisata-derived carbon dots and their anti-depression effect

**DOI:** 10.3389/fmolb.2023.1334083

**Published:** 2024-01-08

**Authors:** Xiaopeng Li, Ertong Dai, Menghan Li, Ruolan Kong, Jinye Yuan, Tingjie Li, Shuxian Wang, Yue Zhang, Hui Kong, Yan Zhao

**Affiliations:** ^1^ School of Traditional Chinese Medicine, Beijing University of Chinese Medicine, Beijing, China; ^2^ Qingdao Eighth People’s Hospital, Qingdao, Shandong, China; ^3^ School of Chinese Materia Medica, Beijing University of Chinese Medicine, Beijing, China; ^4^ School of Life Sciences, Beijing University of Chinese Medicine, Beijing, China

**Keywords:** aurantii fructus immaturus, carbon dots, depression, traditional Chinese medicine, nanomaterial

## Abstract

**Introduction:** Depression is a common illness worldwide. However, the current treatments available for depression only achieve relative success, often come with several side effects, and are associated with high costs. Aurantii Fructus Immaturus (AFI) has a rich historical legacy in Traditional Chinese Medicine (TCM) for its traditional use as a treatment for depression. In this research, our primary objective is to examine the potential antidepressant properties and the mechanisms at play behind a particular bioactive compound found in AFI, which is referred to as carbon dots derived from AFI Carbonisata (AFIC-CDs).

**Methods:** Extracted and isolated the AFIC-CDs from the decoction of AFIC, then characterized the morphological structure and functional groups comprehensively. We then utilized two distinct models to investigate the anti-depressive properties of AFIC-CDs: the chronic unpredictable mild stress (CUMS) model and the reserpine-induced pain-depression dyad model. In the CUMS model, we assessed immobile time and measured neurotransmitter levels in the mouse brain cortex. In the pain-depression dyad model, we evaluated immobile time, neurotransmitter levels, interleukin-1 (IL-1β) and tumor necrosis factor-α (TNF-α) levels, and the expression of mRNA of brain-derived neurotrophic factor (BDNF) and tryptophan hydroxylase 2 (Tph2).

**Results:** AFIC-CDs were found to have abundant chemical groups, and their diameter ranged from 2 to 10 nm. In the CUMS model, AFIC-CDs demonstrated significant effects. They reduced the immobile time of the mice and increased the levels of serotonin (5-HT), dopamine (DA), and norepinephrine (NE) in the mouse brain cortex. In the pain-depression dyad model, the AFIC-CDs groups decreased the immobile time, showed effect in increasing both the neurotransmitters’ levels and the expression of mRNA of BDNF and Tph2, and decreased the IL-1β and TNF-α levels in mouse brain cortex. Taken together, these results strongly indicate that AFIC-CDs possess significant antidepressant activity.

**Conclusion:** AFIC-CDs demonstrate promising therapeutic potential in the treatment of depression, suggesting that they may become a valuable candidate for depression management. This not only extends the understanding of the biological activity of carbon dots (CDs) but also opens up new possibilities for the development of effective depression treatment strategies.

## 1 Introduction

For individuals worldwide, depression remains a prevalent and often inadequately addressed condition, with nearly 280 million people affected. While treatments like SSRIs, TCAs, and psychological interventions exist, their relative success and potential side effects, coupled with the high cost, pose significant challenges, particularly in low- and middle-income countries where access is limited. To address this pressing issue, our research focuses on exploring innovative approaches to alleviate the suffering of individuals with depression. Adding to the complexity, pain and depression are two critical public health issues that frequently coexist, placing a substantial burden on individuals and society. Since these two symptoms frequently occur together and potentially have a common underlying biological basis, although the precise neurobiological mechanisms are not yet fully understood, research indicates a strong connection between pain and depression, and they might be influenced by a shared neural regulatory system (Li, 2015).

In Traditional Chinese Medicine (TCM), qi represents vital energy essential for the proper functioning of organs. Emotional stagnation can lead to a stagnation of qi (Wang et al., 2021a). As a result, the fundamental approach to treating depression in TCM involves the regulation of qi movement (Qiu et al., 2014). Aurantii Fructus Immaturus (AFI) refers to the dried immature fruit of *Citrus aurantium L* or *Citrus sinensis Osbeck*. It is renowned in TCM as a representative remedy for dispersing stagnated liver qi, a condition often associated with depression-like symptoms such as insomnia, pain, feelings of depression, and sadness. AFI has been utilized for over two millennia to alleviate these distressing symptoms.

Carbon dots (CDs) are typically described as quasi-zero-dimensional (quasi-0D) carbon-based nanomaterials. They have found widespread application in biomedical science and have made a significant impact on disease treatment (Li et al., 2022). CDs derived from charcoal in TCM are considered as extracting from natural product. In our study, we utilized CDs obtained from AFI carbonisata (AFIC) and assessed their potential anti-depression effects.

On the previous research of our team, we found that the main bioactive ingredient may be the CDs of the herbs. For example, the Phellodendri Chinensis Cortex-based CDs is the main ingredient of the Phellodendri Chinensis Cortex charcoal for treating psoriasis (Zhang et al., 2021). GRR-CDs, the novel CDs derived from Glycyrrhizae Radix et Rhizoma, which have explicit anti-ulcer activity (Liu et al., 2021). CDs from Cigarette Mainstream may have antianxiety and certain sedative effects (Zhao et al., 2021). AFIC-CDs have anti-hyperuricemic and anti-gouty arthritis activities (Wang et al., 2019).

**FIGURE 1:**
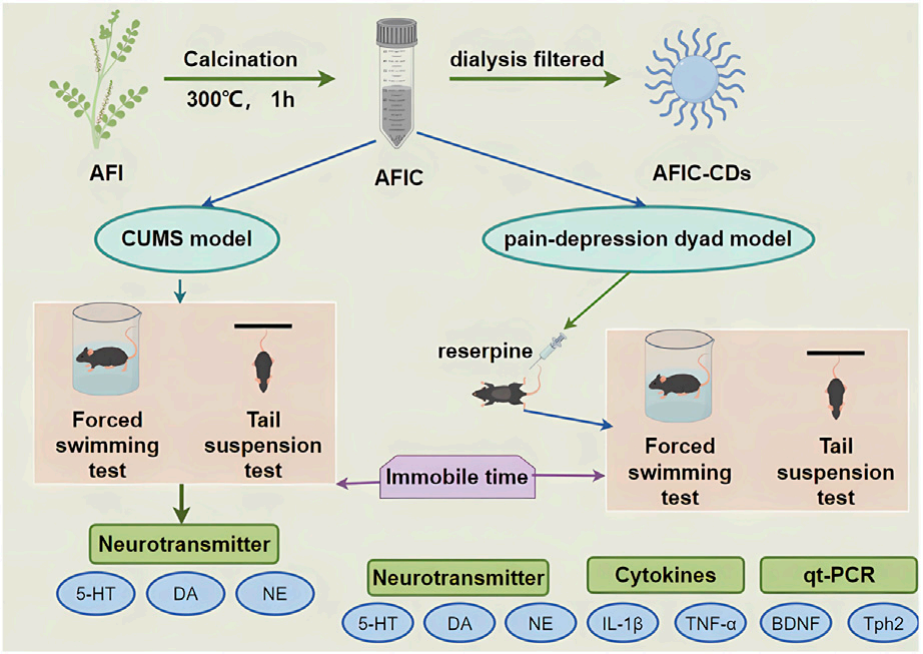
Diagram of the experimental protocol (By Figdraw).

As a result of our findings, we developed a hypothesis suggesting that AFIC-CDs may possess antidepressant properties. To explore this hypothesis, we established a depression model using both the CUMS model and a pain-depression dyad model, Figure 1 illustrating the key steps in our research.

## 2 Materials and methods

### 2.1 Materials

The AFI was procured from Beijing Qiancao Traditional Chinese Medicine Co, Ltd, located in Beijing, China. The reserpine drugs utilized in this study were acquired from Kanglang Biological Technology in Shanghai, China. In the process of preparing the reserpine solution, reserpine was initially dissolved in 0.5% glacial acetic acid and subsequently diluted to achieve a concentration of 1 mg/mL using distilled water. The fluoxetine solution was prepared by dissolving fluoxetine hydrochloride dispersible tablets to achieve a concentration of 0.2 mg/mL. The TNF-α and IL-1β ELISA kits were acquired from R&D Systems (United States). Dialysis membranes (MWCO: 1,000 Da) were obtained from Beijing Ruida Henghui Technology Development Co., Ltd. (Beijing, China). Cell counting kit-8 (CCK-8) were brought from Beijing Bairuiji Biotechnology Co., Ltd. (Beijing, China). Dulbecco’s Modified Eagle Medium (DMEM) and fetal bovine serum (FBS) were purchased from Corning Co., Ltd. (New York, United States).

### 2.2 Animals

Fifty-two male adult and fifty-two female special pathogen-free (SPF) Kunming mice (obtained from the Laboratory Animal Center, Si Beifu) weighing (30 ± 2) g, were employed in the study. All animal procedures were conducted in strict compliance with the guidelines established by the Animal Experimentation of Beijing University of Chinese Medicine and received approval from the committee. The mice were accommodated in cages under controlled laboratory conditions, including a 12/12-h light/dark cycle, a temperature of 23.0°C ± 2.0°C, and relative humidity maintained at 50%–60%. And they were provided with free access to food and water.

### 2.3 Preparation of AFIC-CDs

**FIGURE 2:**
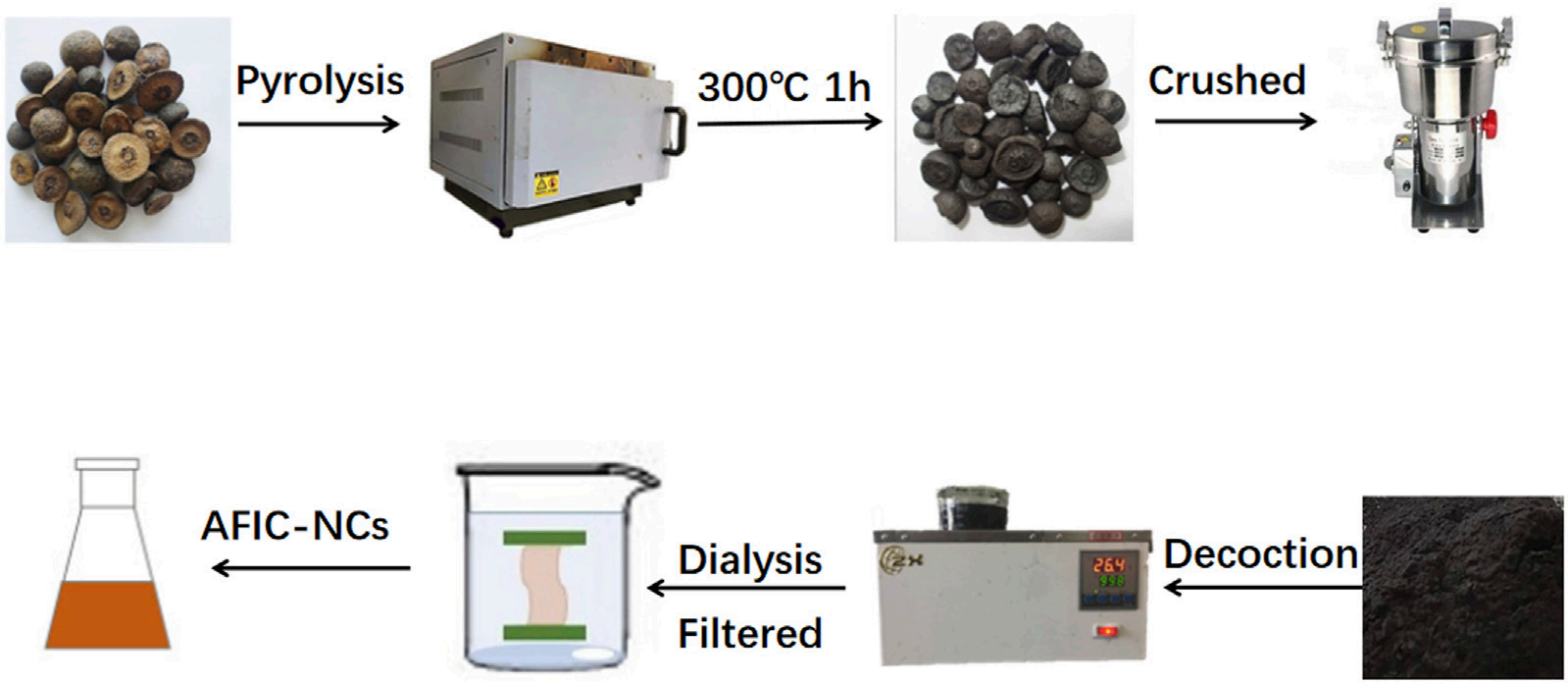
The flowchart for the preparation process of carbon dots derived from Aurantii fructus immaturus carbonisata.

The AFIC-CDs were prepared in our laboratory. 60 g AFI was positioned in a crucible and subjected to heating within a preheated furnace set to 300°C. After it had cooled down naturally, it was subsequently pulverized into a fine powder. The resulting black powder was then dispersed in deionized water and subjected to two rounds of boiling in a water bath, each lasting for 1 h (Luo et al., 2021; Mou et al., 2023). Larger products were removed using a 0.22 μm microporous membrane. Concentrate the filtrates to a total volume of 60 mL, yielding an AFIC solution with an original drug concentration of 1.0 g/mL. Place the AFIC solution into a dialysis bag (MWCO = 1000 D), secure both ends of the dialysis bag with clamps to prevent leakage, and immerse it in deionized water for dialysis. Change the water at least every 8 h during the dialysis, with a total duration exceeding 72 h. Ensure completion of dialysis when the solution outside the dialysis bag appears colorless and transparent. It was then preserved at 4°C for future utilization. Figure 2 illustrates the preparation process of AFIC-CDs.

### 2.4 Characterization of AFIC-CDs

We employed various analytical techniques to examine the particle size and microscopic structure of AFIC-CDs. The transmission electron microscope (TEM; Tecnai G2; FEI Company, Hillsboro, OR, United States) was used for observing particle size and microscopic morphology. For a closer look at structural details and atomic lattice fringes, a high-resolution TEM (HRTEM) system (JEN-1230, Japan Electron Optics Laboratory, Tokyo, Japan) was utilized. To assess photoluminescent and ultraviolet-visible (UV-vis) absorption properties, we recorded spectra with a fluorescence (FL) spectrophotometer (F-4500, Tokyo, Japan) and a UV-vis spectrometer (CECIL, Cambridge, United Kingdom), respectively. The identification of functional groups and the elemental composition of AFIC-CDs was accomplished using an FTIR spectrophotometer (Thermo Fisher, CA, United States) and X-ray photoelectron spectroscopy (XPS, Thermo Fisher Scientific, MA, United States). Furthermore, the crystalline nature of AFIC-CDs was investigated through X-ray diffraction (XRD) using a D8 Venture Plus X-ray Diffractometer (Bruker AXS, Karlsruhe, Germany). The main components in the solution of AFIC and AFIC-CDs were identified using high-performance liquid chromatography (HPLC; Agilent 1,260) with the detection wavelength at 284 nm.

### 2.5 The chronic unpredictable mild stress model

To evaluate the effect of AFIC-CDs to treat depression, we used the CUMS depression model, including forced swimming (Porsolt et al., 1977) and tail suspension (Steru et al., 1985) models. Male SPF mice (*n* = 30) were randomized into the following five groups (*n* = 6): Normal saline solution (NS); Positive control (Fluoxetine, 2 mg/kg); High (8 mg/kg), Medium (4 mg/kg) and Low (2 mg/kg) doses of AFIC-CDs. In the control group, mice received intragastric administration of 0.3 mL of saline water. In the other groups, equivalent volume solutions were administered via intragastric administration, following the same procedure as in the control group. The intragastric drug administration took place 1 h before subjecting the mice to the CUMS model.

#### 2.5.1 Forced swimming test

The experimental procedure involved immersing individual mice in a 2000 mL beaker filled with water to a depth of 15 cm, with the water temperature maintained at 25°C. The immersion lasted for a total of 6 min, with the initial 1 min designated as the adaptation period. The immobility time was calculated during the final 5 min of this period. During this time, if a mouse exhibited a lack of movement and remained floating in the water without any noticeable struggle or only displayed minimal movements necessary to stay afloat, it was considered to be in an immobile state. This approach allowed for the measurement of immobility as a relevant parameter in the experiment.

#### 2.5.2 Tail suspension test

The mice were affixed with adhesive tape approximately 2 cm from the tip of their tails and placed within an isolated box, designed to provide acoustic and visual isolation. This setup induced a brief and inescapable stress response, leading to an immobile posture. Immobility was defined as complete passivity and motionlessness. Immobility time was recorded over a 6-min period, with the last 4 min used for calculation, following an initial 2-min adaptation period. This method assessed immobility as an indicator of the stress response.

### 2.6 The reserpine-induced pain-depression dyad model

The mice were subjected to a pain-depression dyad model by receiving hypodermic injections of reserpine (Arora et al., 2011). That is, mice accepted hypodermic injection with reserpine at a dose of the 1 mg/kg, once a day for 3 consecutive days. Control group mice received hypodermic injections of the same volume of normal saline, also once a day for 3 days. Female SPF mice (*n* = 30) were randomized divided into the five groups (*n* = 6): Normal saline solution (NS); Model (NS); High (8 mg/kg), Medium (4 mg/kg) and Low (2 mg/kg) doses of AFIC-CDs. Intragastric drug administration was carried out on the same day as pain-depression dyad modeling, with dosing occurring twice a day for three consecutive days. In the control and model groups, mice received intragastric administration of 0.3 mL of saline water. The other groups were administered an equivalent volume of the solution as mentioned above. After hypodermic injection with reserpine, the mice were exposed to CUMS. Evaluate the antidepressant activity of AFIC-CDs by calculating the immobile time.

### 2.7 Specimen extraction

Upon the completion of the experiment, the mice were promptly subjected to decapitation. The brains were subsequently excised on an ice-cold surface, and the cortex was isolated and weighed. The cortex was then homogenized with a PBS solution. The resulting homogenate was transferred to a centrifuge tube and centrifuged at 10,000 rpm and 4°C for a duration of 10 min. Following centrifugation, the supernatant was meticulously collected and preserved in a freezer at −80°C for future utilization.

### 2.8 Assessing the neurotransmitter levels and cytokines levels

The levels of 5-HT, NE, DA, TNF-α, and IL-1β in the brain cortex were determined via enzyme-linked immunosorbent assay (ELISA) using kits obtained from Uscn Life Science Inc., located in Wuhan, China, and following the manufacturer’s instructions. The absorbance at 450 nm was measured with a microplate reader (Biotek, VT, United States), and the neurotransmitter levels were quantified based on standard curves.

### 2.9 Quantitative real-time polymerase chain reaction (qRT-PCR) to assess the BDNF and Tph2

Total RNAs (1 μg) were extracted from the brain cortex, and then isolated with Trizol reagent. The Synthesis Kit and realtime fluorescence quantitative PCR Kit were from Solarbio (Beijing). The primers used were as follows: 5′-AACTAGAGGATGTGCCGTGG-3′and 5′-CCT​TGA​ATC​CTG​GGT​GGT​CG-3′ (Tph2),5′-AACTCCCAGTGCCGAACTACCC-3′and 5′-TCC​TTA​TGA​ATC​GCC​AGC​CAA​TTC​TC-3′ (BDNF).

### 2.10 Cytotoxicity assay of AFIC-CDs

In order to assess the hazards of AFIC-CDs and investigate its safety, the CCK-8 experiments were performed to recognize the cytotoxicity of AFIC-CDs to human LO2 hepatocyte. The cells were cultured in DMEM medium containing 10% fetal bovine serum (FBS) in a humidified 5% CO2 atmosphere at 37°C. Subsequently, the LO2 cells were seeded in a 96-well plate at a density of 1 × 105 cells per 100 μL/well and incubated for 24 h, respectively. Then, different concentrations of AFIC-CDs (39.06, 78.13, 156.25, 312.5, 625, 1,250, 2,500, 5,000 and 10,000 μg/mL) were added to the designated wells for 48 h and the control cells were treated with DMEM medium. After these plates were washed thrice with PBS, 10 μL of CCK-8 is added to the plate and incubated for 4 h. Additionally, a microplate reader (Biotek, Vermont, United States) was utilized to record the absorbance of each well. Finally, the cell viability (%) calculation formula is as follows:

$$Cell\;viability\left(\%\;of\;control\right)=\frac{\text{Ae}-\text{Ab}}{\text{Ac}-\text{Ab}}\times 100$$

Ae, Ab and Ac represent the absorbance at a 450 nm wavelength (A450) of the experimental, blank and control groups, respectively.

### 2.11 Biosafety experiment in mice

The experiment involved a total of 44 specific-pathogen-free (SPF) kunming healthy mice, evenly distributed between male and female, with a weight of 30 ± 2 g. The mice were randomly divided into two groups: the maximum dose group receiving AFIC-CDs (400 mg/kg) and the normal control group, each consisting of 22 mice. The animals were weighed and marked accordingly. In the maximum dose group, mice were administered AFIC-CDs via gavage at the highest drug concentration of 10 mg/mL, with a maximum volume of 0.4mL/10g, which the mice could tolerate. The normal control group received an equivalent dose of physiological saline. The general condition and behavioral performance of the mice were observed post-administration.

Daily administration and continuous observation were carried out for 14 days. On the first, fourth, and seventh days post-administration, abdominal dissections were performed. Four mice from each experimental and control group were sacrificed each time. On the 14th day, the remaining mice were euthanized, with 10 mice from each experimental and control group. Approximately 1 mL of blood was collected from the eyeballs before euthanasia, allowed to stand for 2 h, centrifuged (3,000 rpm, 10 min), and the supernatant was collected and stored at −80°C for subsequent use. Blood routine parameters (WBC, RBC, HGB, PLT), blood biochemical indicators (ALT, AST, BUN, and CRE values) were analyzed. Euthanasia was performed by cervical dislocation, and *postmortem* examinations were conducted by dissecting major organs (heart, liver, spleen, lungs, and kidneys). The organs were fixed in 4% paraformaldehyde, subjected to hematoxylin-eosin (HE) staining, and examined for pathological changes.

### 2.12 Statistical analysis

The experimental results were analyzed using the Statistical Package for the Social Sciences (SPSS, version 22.0). Normally distributed data were presented as means ± standard deviation, and analyzed using one-way analysis of variance (ANOVA). For non-normally distributed data, medians ± interquartile range were used, and the Kruskal–Wallis test was applied for analysis. To compare data between two groups, the Wilcoxon rank test was utilized. Statistical significance was defined as *p* < 0.05 and *p* < 0.01.

## 3 Results

### 3.1 Characterization of AFIC-CDs

**FIGURE 3:**
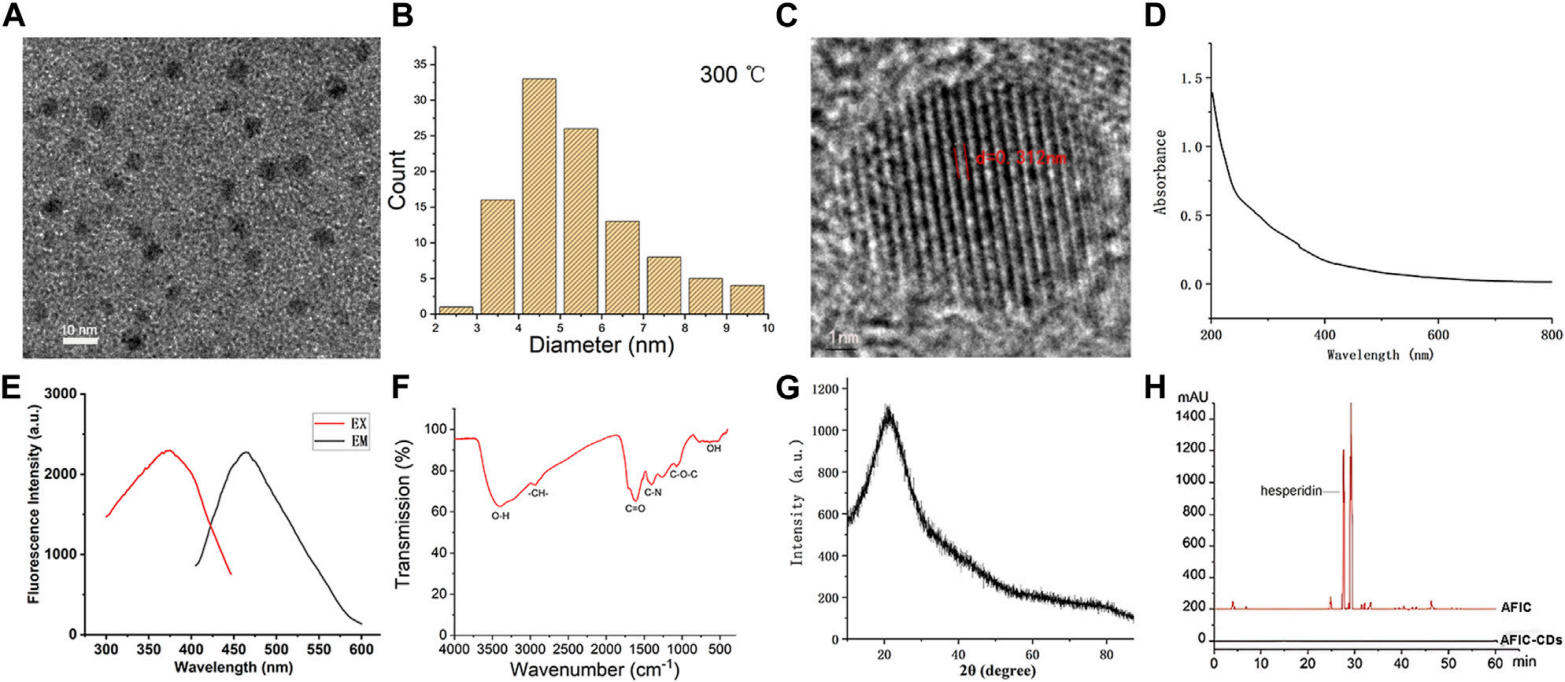
Characterization of AFIC-CDs. **(A)** TEM images of AFIC-CDs displaying ultra-small particles. **(B)** histogram depicting particle size distribution. **(C)** High-resolution TEM image of AFIC-CDs. **(D)** UV-visible spectra of the AFIC-CDs. **(E)** Excitation and emission fluorescence spectra. **(F)** The Fourier-transform infrared spectrum of AFIC-CDs. **(G)**X-ray diffraction pattern of AFIC-CDs. **(H)** The high-performance liquid chromatography fingerprint spectra of AFIC and AFIC-CDs.

As depicted in Figure 3A, the TEM image shows that the AFIC-CDs were spherical, monodispersed and the particle size distribution was concentrated in the range of 2–10 nm (Figure 3B). In addition, the HRTEM images indicated that the lattice distance of AFIC-CDs was 0.313 nm (Figure 3C). The UV-Vis absorption spectrum revealed that AFIC-CDs had a broad spectrum without clear peaks (Figure 3D). The fluorescence spectra of the AFIC-CDs showed a maximum emission and excitation at 467 and 374 nm, respectively (Figure 3E).

The surface chemical characteristics of AFIC-CDs were characterized by FTIR spectroscopy (Figure 3F). The absorption peak at 3,416 cm^−1^ may be the stretching vibration peak of -OH. The presence of C-H group was detected at 2,950 cm^−1^. The absorption peak at 1,607 cm^−1^ indicated the characteristic absorption of C=O. Furthermore, the peak existing at 1,402 cm^−1^ was identified as C-N. The absorption peak at 1,255 cm^−1^ may be correspond to C-O-C. The weak absorption signals at 761 cm^−1^ were related to O-H stretching. The wide-range XRD pattern in Figure 3G shows that there is a diffraction peak at approximately 22.765°, consistent with the results obtained from HRTEM. And as shown in Figure 3H, the HPLC results indicated the presence of compounds, such as hesperidin, in the AFIC solution. However, the characteristic peak of this component in AFIC was notably absent in the AFIC-CDs solution. This absence provides additional evidence that active small molecule compounds were not present in AFIC-CDs.

**FIGURE 4:**
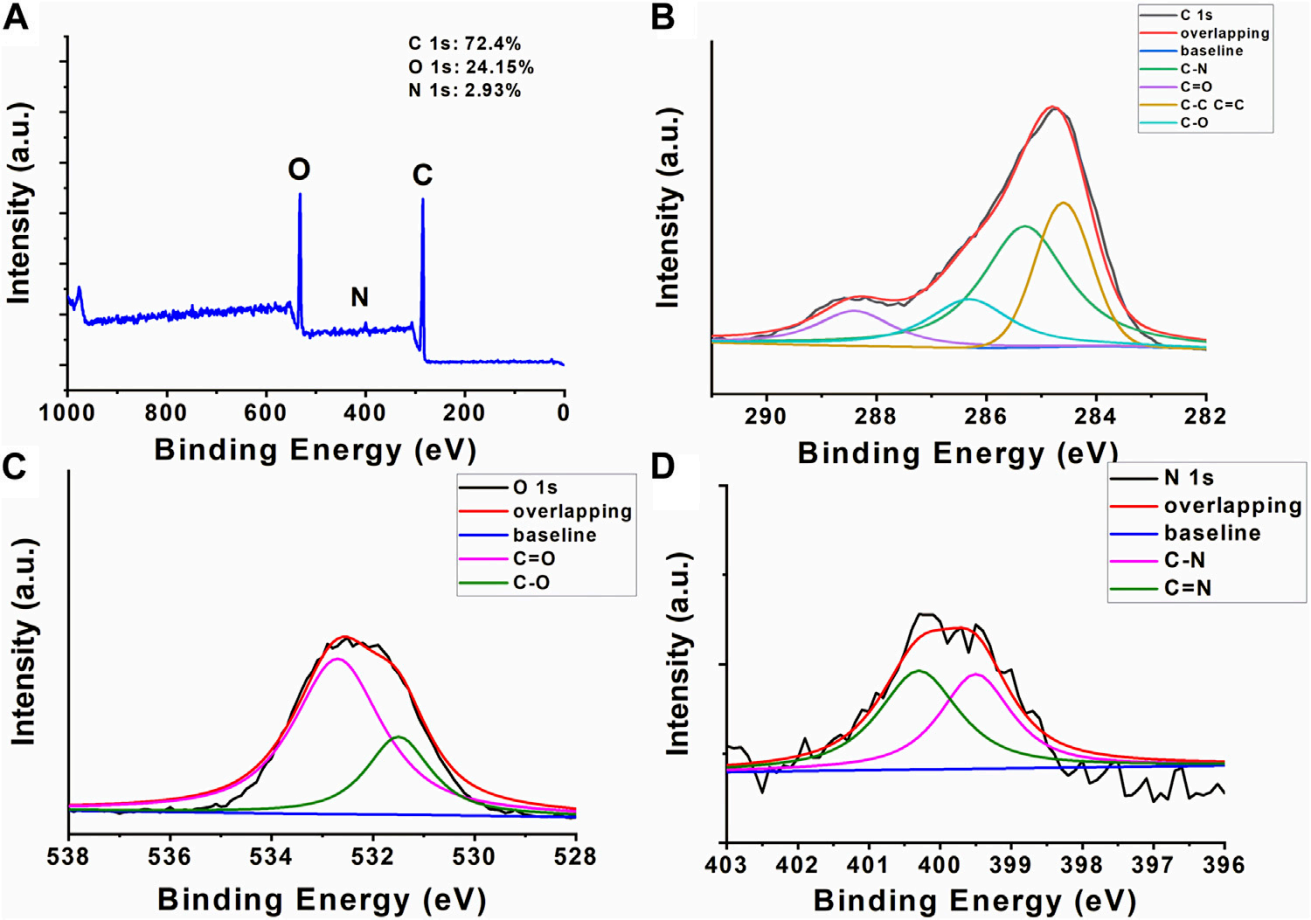
X-ray photoelectron spectroscopy of AFIC-CDs. **(A)** The full-scan x-ray photoelectron spectroscopy spectra of AFIC-CDs which contain 72.4% of carbon, 24.15% of oxygen and 2.93% nitrogen. High-resolution x-ray photoelectron spectroscopy spectra of **(B)** carbon, **(C)** oxygen and **(D)** nitrogen peaks are collected to illustrate the detailed bonding formation of AFIC-CDs.

XPS analysis (Figure 4) was employed to determine the surface element composition and microchemical environment of AFIC-CDs. As shown in Figure 4, the CDs exhibit three dominant peaks corresponding to carbon (C) at 72.4%, oxygen (O) at 24.15%, and nitrogen (N) at 2.93%. The full-scan XPS spectrum of AFIC-CDs clearly reveals three characteristic peaks at 284.6 eV, 399.7 eV, and 531.8 eV, which are attributed to the binding energy signals of C 1s, N 1s, and O 1s, respectively. The C 1s spectrum (Figure 4B) can be deconvoluted into four distinct component peaks, each associated with specific chemical states: 284.6 eV (sp2 C), 286.3 eV (C-O), 285.3 eV (C-N), and 288.4 eV (C=O). Similarly, the O 1s spectrum (Figure 4C) comprises two component peaks, likely corresponding to 531.5 eV (C-O) and 532.7 eV (C=O). The N 1s spectrum (Figure 4D) predominantly consists of two subpeaks at 399.5 eV and 400.3 eV, corresponding to C-N and N-H, respectively.

### 3.2 The chronic unpredictable mild stress model

#### 3.2.1 The tail suspension and forced swimming tests results

**FIGURE 5:**
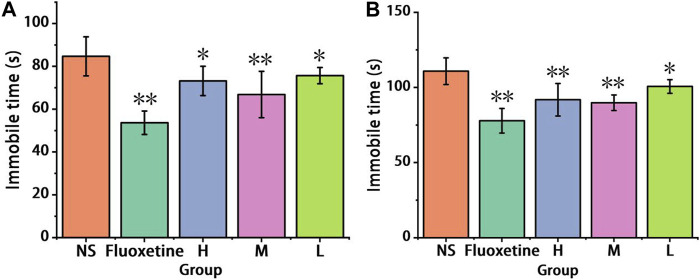
Effect of antidepressant of AFIC-CDs on CUMS model. **(A)** Immobile time in tail suspension test. **(B)** Immobile time in forced swimming test. Mice were assigned into five groups: control (Normal saline solution [NS]), Positive control (Fluoxetine, 2 mg/kg), different doses of AFIC-CDs groups (High [H]: 8 mg/kg; Medium [M]: 4 mg/kg; Low [L]: 2 mg/kg). Data were expressed as means ± standard deviation (SD). *p < 0.05,**p < 0.01 vs. control group.

In the tail suspension test, as depicted in Figure 5A, the immobility time exhibited a significant reduction (*p* < 0.01) in the Fluoxetine group (53.67 ± 5.47 s), the medium-dose group (66.83 ± 10.82 s), as compared to the control group (84.67 ± 9.1 s), demonstrating a clear antidepressant effect. Both the high-dose (73.17 ± 6.85 s) and low-dose (75.67 ± 3.78 s) groups also showed a reduction in immobility time (*p* < 0.05), indicating antidepressant activity. These results strongly suggest that AFIC-CDs possess antidepressant properties.

In the forced swimming test, as depicted in Figure 5B, the immobility time exhibited a significant decrease (*p* < 0.01) in the high and medium dose groups (91.83 ± 10.83 s, 89.83 ± 5.19 s) as well as the Fluoxetine group (77.83 ± 8.18 s), in comparison to the control group (110.83 ± 8.84 s). Additionally, the low dose group (100.67 ± 4.55 s) also demonstrated a reduction in immobility time (*p* < 0.05). These results strongly support the effectiveness of AFIC-CDs in combating depression.

#### 3.2.2 Neurotransmitter levels

**FIGURE 6:**
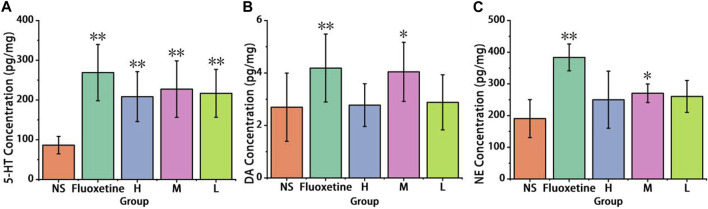
Effect of antidepressant of AFIC-CDs on neurotransmitter levels. **(A)** Effect of AFIC-CDs on 5-HT levels. **(B)** Effect of AFIC-CDs on DA levels. **(C)** Effect of AFIC-CDs on NE levels. Mice were assigned into five groups: control (Normal saline [NS]), Positive control (Fluoxetine, 2 mg/kg), different doses of AFIC-CDs groups (High [H]: 8 mg/kg; Medium [M]: 4 mg/kg; Low [L]: 2 mg/kg). Data were expressed as means ± standard deviation (SD). *p < 0.05,**p < 0.01 vs. control group.

As illustrated in Figure 6A, a significant increase in brain cortex 5-HT levels was observed in the Fluoxetine group (269.07 ± 70.87 pg/mg), as well as in the high-dose group (208.5 ± 62.78 pg/mg), medium-dose group (227.47 ± 71.09 pg/mg), and low-dose group (216.83 ± 60.17 pg/mg), when compared to the control group (86.39 ± 21.95 pg/mg) (*p* < 0.01). Notably, the medium-dose group exhibited a particularly pronounced increase.

As shown in Figures 6A, B significant improvement in brain cortex DA levels was observed in the Fluoxetine group (4.19 ± 1.29 pg/mg) and the medium-dose group (4.04 ± 1.12 pg/mg) when compared to the control group (2.7 ± 1.3 pg/mg) (*p* < 0.01). These results indicate that AFIC-CDs have the ability to elevate the levels of DA in the brain cortex.

As shown in Figure 6C, compared with the control group (190.43 ± 59.85 pg/mg), the Fluoxetine group (383.67 ± 42.2 pg/mg), medium-dose group (270.58 ± 29.23 pg/mg) show increase in NE levels (*p* < 0.01). These findings suggest that AFIC-CDs can enhance NE levels in the brain cortex of mice.

### 3.3 The reserpine induced pain-depression dyad model

#### 3.3.1 Tail suspension and forced swimming test results

After a hypodermic injection with reserpine, the immobility time of the mice was assessed in both the tail suspension test and the forced swimming test.

**FIGURE 7:**
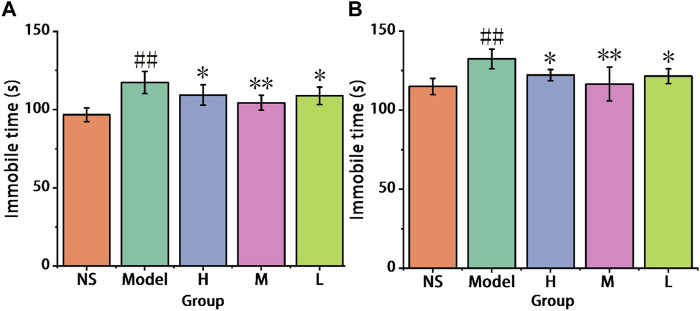
Effect of AFIC-CDs on chronic unpredicted mild stress paradigms in reserpine induced comorbid pain and depression mice. **(A)** Immobile time in tail suspension test. **(B)** Immobile time in forced swimming test. Mice were assigned into five groups: control (Normal saline [NS]), Model, different doses of AFIC-CDs groups (High [H]: 8 mg/kg; Medium [M]: 4 mg/kg; Low [L]: 2 mg/kg). Data were expressed as means ± standard deviation (SD). *p < 0.05,**p < 0.01 vs. Model group. ##*p* < 0.01 vs. control group.

In the tail suspension test, as depicted in Figure 7A, chronic administration of reserpine resulted in an increase in immobility time in the model group (117.33 ± 7.12 s) compared to the control group (96.67 ± 4.32 s). The high-dose group (109.33 ± 6.44 s) and low-dose group (108.83 ± 5.49 s) that received AFIC-CDs exhibited a reduction in immobility time compared to the model group, demonstrating anti-depressive activity (*p* < 0.05). Particularly noteworthy were the results of the medium-dose group (104.33 ± 4.72 s), which showed a significant reduction in immobility time (*p* < 0.01).

In the forced swimming test, as shown in Figure 7B, chronic administration of reserpine led to an increase in immobility time in the model group (132.33 ± 6.25 s) compared to the control group (115 ± 5.10 s). Notably, the medium-dose group (116.5 ± 10.67 s) exhibited a significant decrease in immobility time compared to the model group, demonstrating a clear antidepressant effect (*p* < 0.01). The high-dose group (122.17 ± 3.54 s) and low-dose group (121.5 ± 4.64 s) also showed reduced immobility time (*p* < 0.05). These results indicate that, in the reserpine-induced pain and depression model, AFIC-CDs exhibit significant antidepressant activity.

#### 3.3.2 Neurotransmitter levels

**FIGURE 8:**
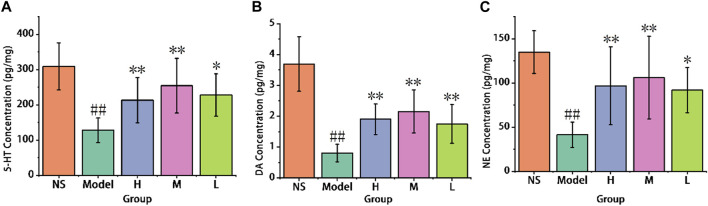
Effect of AFIC-CDs on neurotransmitter levels in reserpine-induced pain-depression dyad mice. **(A)** Effect of AFIC-CDs on 5-HT levels. **(B)** Effect of AFIC-CDs on DA levels. **(C)** Effect of AFIC-CDs on NE levels. Mice were assigned into five groups: control (Normal saline [NS]), Model, different doses of AFIC-CDs groups (High [H]: 8 mg/kg; Medium [M]: 4 mg/kg; Low [L]: 2 mg/kg). Data were expressed as means ± standard deviation (SD). *p < 0.05,**p < 0.01 vs. Model group. ##*p* < 0.01 vs. control group.

As shown in Figure 8A, chronic administration of reserpine led to a reduction in 5-HT levels in the model group (127.72 ± 35.16 pg/mg) compared to the control group (308.85 ± 66.30 pg/mg). And comparison with the model group, the high (213.11 ± 63.75 pg/mg) and medium-dose (254.32 ± 77.52 pg/mg) of AFIC-CDs groups show significant improvement (*p* < 0.01). These results suggest that AFIC-CDs increased 5-HT levels in the mouse brain cortex, and the antidepressant effectiveness of AFIC-CDs may be associated with the promotion of elevated 5-HT levels.

As illustrated in Figure 8B, the reserpine-induced pain-depression dyad model group displayed a significant decrease in DA levels when compared to the control group (0.81 ± 0.28 vs. 3.69 ± 0.88 pg/mg). In contrast, the high-dose (1.9 ± 0.5 pg/mg), medium-dose (2.15 ± 0.7 pg/mg), and low-dose (1.75 ± 0.63 pg/mg) groups of AFIC-CDs exhibited a significant increase in DA levels (*p* < 0.01) when compared to the model group (0.81 ± 0.28 pg/mg). These results suggest that AFIC-CDs have an effect on increasing the levels of dopamine in the mouse brain cortex.

As shown in Figure 8C, the model group exhibited a significant decrease in NE levels in the mouse brain cortex when compared to the control group (41.43 ± 14.34 pg/mg vs. 134.87 ± 24.09 pg/mg). Conversely, the high-dose group (96.73 ± 44.01 pg/mg) and the medium-dose group (106.05 ± 46.7 pg/mg) both showed a significant increase in NE levels when compared to the model group (*p* < 0.01). These results indicate that AFIC-CDs produced a significant improvement in NE levels in the mouse brain cortex.

#### 3.3.3 Cytokines levels

**FIGURE 9:**
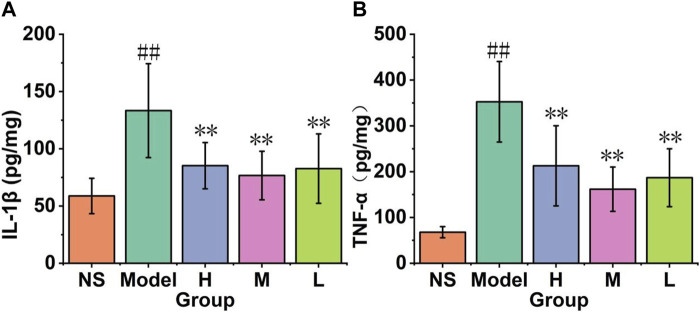
Effect of AFIC-CDs on cytokines levels in reserpine-induced pain-depression dyad mice. **(A)** Effect of AFIC-CDs on IL-1β levels. **(B)** Effect of AFIC-CDs on TNF-α levels. Mice were assigned into five groups: control (Normal saline [NS]), Model, different doses of AFIC-CDs groups (High [H]: 8 mg/kg; Medium [M]: 4 mg/kg; Low [L]: 2 mg/kg). Data were expressed as means ± standard deviation (SD). *p < 0.05,**p < 0.01 vs. Model group. ##*p* < 0.01 vs. control group.

As shown in Figure 9A, there was a significant increase in the IL-1β level in the reserpine administered model mice as compared to the control group (58.77 ± 15.44 pg/mg). Comparison with the model group (133.29 ± 41.06 pg/mg), the high (85.22 ± 20.2 pg/mg), medium (76.65 ± 21.22 pg/mg) and low (82.68 ± 30.34 pg/mg) doses groups show significant decreased in brain IL-1β level (*p* < 0.01). That means AFIC-CDs can inhibit the level of IL-1β in the brain, especially in the medium-dose group.

As shown in Figure 9B, the model group exhibited a significant increase in brain TNF-α levels compared to the control group (352.67 ± 87.95 pg/mg vs. 67.7 ± 12.17 pg/mg). However, when compared with the model group, the high-dose group (212.79 ± 87.57 pg/mg), medium-dose group (161.77 ± 48.31 pg/mg), and low-dose group (186.83 ± 63.23 pg/mg) all demonstrated a significant decrease in brain TNF-α levels (*p* < 0.01). These findings indicate that AFIC-CDs have the capacity to effectively inhibit TNF-α levels in the brain, with particularly noteworthy effects observed in the medium-dose group.

#### 3.3.4 The results of the qRT-PCR analysis

**FIGURE 10:**
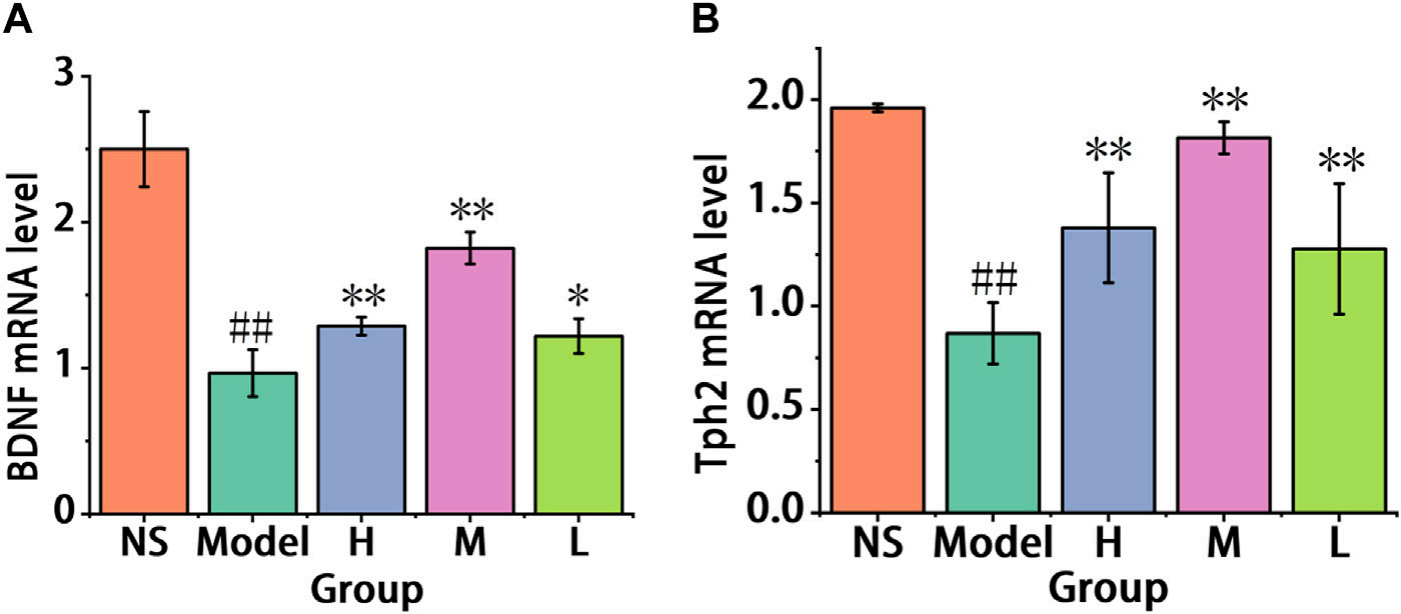
The results of qRT-PCR analysis. **(A)** Effect of AFIC-CDs on the express of brain cortex BDNF mRNA levels. **(B)** Effect of AFIC-CDs on the express of brain cortex Tph2 mRNA level. Mice were assigned into five groups: control (Normal saline [NS]), Model, different doses of AFIC-CDs groups (High [H]: 8 mg/kg; Medium [M]: 4 mg/kg; Low [L]: 2 mg/kg). Data were expressed as means ± standard deviation (SD). *p < 0.05,**p < 0.01 vs. Model group. ##*p* < 0.01 vs. control group.

As shown in Figure 10A, the reserpine-induced pain-depression dyad model mice (0.97 ± 0.16) showed a significant decrease in the expression of BDNF mRNA in the brain cortex compared to the control group (2.5 ± 0.26). In comparison with the model group, the low-dose group (1.22 ± 0.12) exhibited an increase (*p* < 0.05), while the high-dose group (1.29 ± 0.06) and the medium-dose group (1.82 ± 0.1) showed a significant increase (*p* < 0.01). These results indicate that all doses of AFIC-CDs groups increase the BDNF mRNA levels in the mouse brain cortex, with the medium-dose group showing the most significant enhancement.

As illustrated in Figure 10B, comparison with the control group (1.96 ± 0.02), the reserpine-induced pain and depression model (0.87 ± 0.15) mice show significant decrease in brain cortex Tph2 mRNA level. The high (1.38 ± 0.27), medium (1.81 ± 0.079) and low (1.28 ± 0.32) doses groups show significant increase (*p* < 0.01). These results indicate that AFIC-CDs increased the Tph2 mRNA level in the brain cortex, and the brain cortex 5-HT level may be influenced by the increase of the express of Tph2 mRNA.

#### 3.4.1 Biosafety evaluation

**FIGURE 11:**
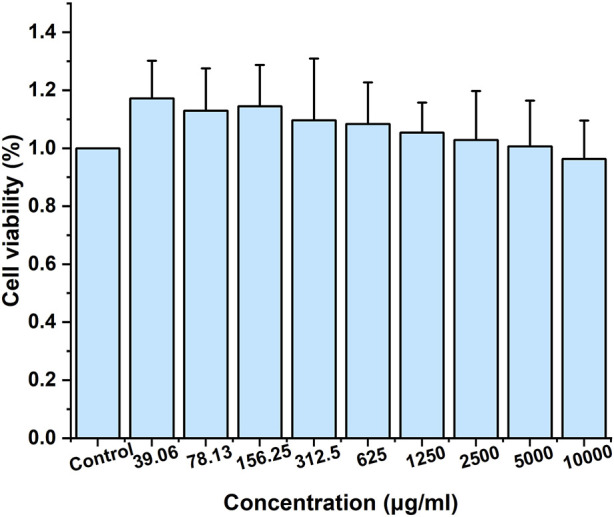
Effect of different concentrations of AFIC-CDs on the viability of LO2 cells via CCK-8 assay for 48 h.

Given the significant therapeutic potential of carbon dots (CDs), there is considerable concern regarding their safety. Therefore, it is essential to assess their biosafety before incorporating them into clinical studies. As shown in Figure 11, at concentrations ranging from 39.06 to 10000 μg/mL, AFIC-CDs demonstrated minimal impact on cell viability. There was no significant inhibitory effect on the proliferation of LO2 cells, indicating that AFIC-CDs exhibit no apparent cytotoxicity towards LO2 cells. These findings suggest that AFIC-CDs are a safe and non-toxic component with excellent biocompatibility.

**FIGURE 12:**
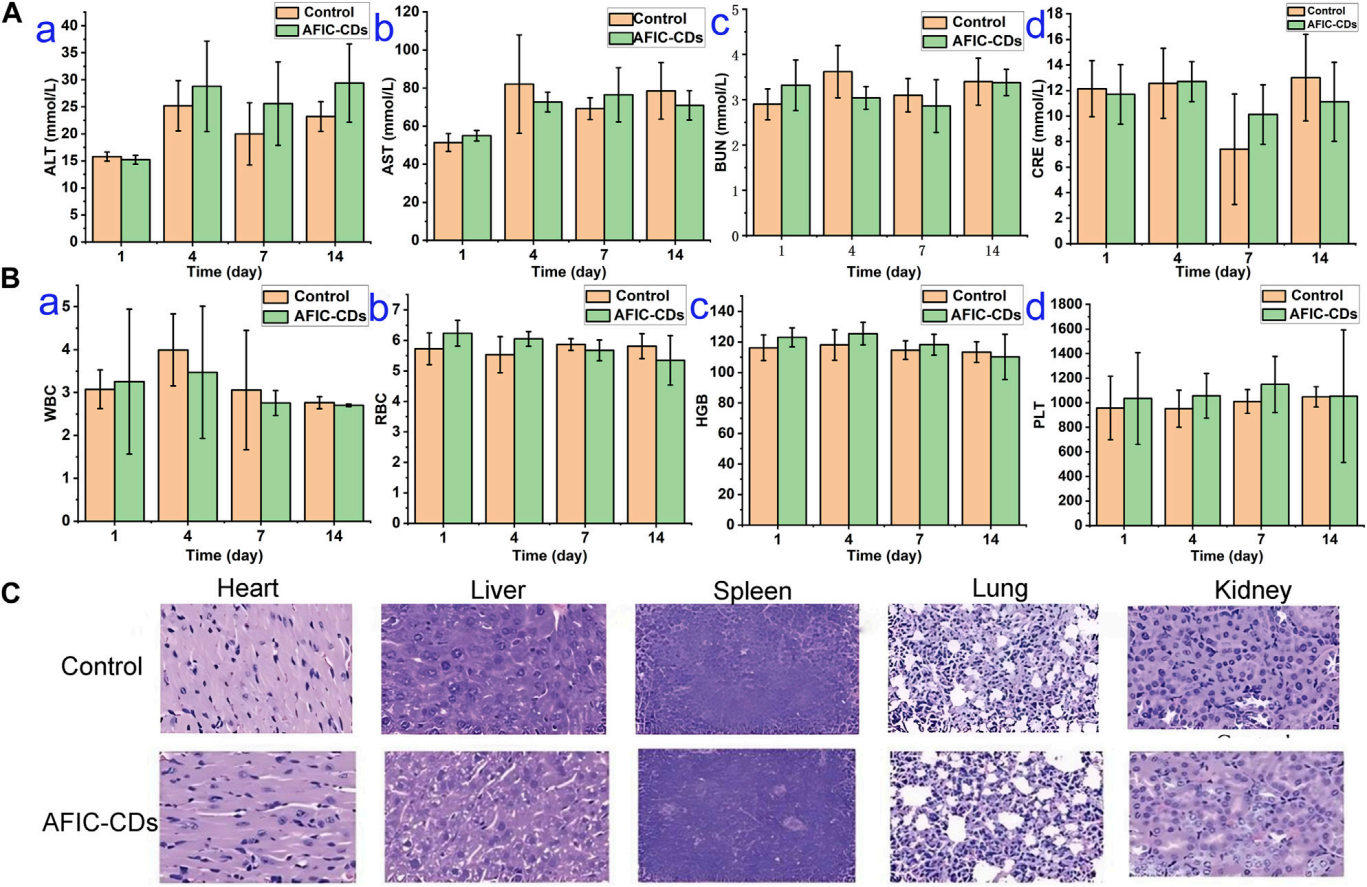
Biosafety experiment of AFIC-CDs. **(A)** Effects of AFIC-CDs on blood biochemistry in mice: ALT **(a)**, AST **(b)**, BUN **(c)** and CRE **(d)**. **(B)** Effects of AFIC-NCs on blood routine in mice: WBC **(a)**, RBC **(b)**, HGB **(c)**, PLT **(d)**. **(C)** Histological evaluation of five major organs of mice in different groups.

Blood chemical examinations, encompassing ALT, AST, BUN, and CRE levels, along with routine blood tests measuring WBC, RBC, HGB, and PLT levels, were performed on mice orally administered with AFIC-CDs. As illustrated in Figure 12, the results indicated an absence of any discernible abnormalities in these parameters. This suggests that the administration of AFIC-CDs had no apparent adverse effects on liver and kidney functions, as well as on the overall composition of the blood. Additionally, we performed a comprehensive evaluation of potential toxicity towards multiple organs *in vivo*. Histological analysis and H&E staining revealed the absence of any morphological or pathological abnormalities in any of the treatment groups. These results strongly suggest that the intervention with AFIC-CDs had minimal impact on the major organs of mice, indicating a favorable safety profile.

## 4 Discussion

Depression, a prevalent and intricate mental disorder, ranks among the leading global causes of disability, known for its high recurrence rate. At its most severe, depression can tragically lead to thoughts of suicide. Individuals grappling with depression often experience persistent low mood, including irritability, feelings of sadness, and emptiness. They may also lose interest in previously enjoyed activities. During depressive episodes, patients may grapple with excessive guilt, profound fatigue, low energy levels, and difficulties concentrating. The complex etiology of depression involves a multifaceted interaction of biological, social, familial, and psychological factors. In TCM, depression is often attributed to the stagnation of liver qi and spleen deficiency, leading to the use of qi-regulating medications such as AFI for its treatment.

Carbon dots (CDs), as a kind of nanomaterials with unique advantages, have displayed substantial potential for applications in the biomedical field, including biological imaging and tumor therapy. Currently, much research focuses on exploring disease treatment and expanding application domains while giving insufficient attention to the choice of raw materials. Compared to CDs derived through chemical processes, CDs obtained from Chinese herbal medicine offer distinct advantages such as an abundant supply of raw materials, straightforward preparation methods, excellent biocompatibility, high water solubility, low toxicity, and cost-effectiveness. They represent an ideal choice as precursor materials for CDs. Significantly, Chinese herbal medicine contains a diverse array of active components, serving as a direct reservoir of heteroatoms. As a result, the investigation into the biological properties of CDs originating from Chinese herbal medicine has garnered substantial attention from numerous researchers, both nationally and internationally, in recent years. (Zhao et al., 2023).

The tail suspension test and forced swimming test are widely recognized tools for evaluating antidepressant activity, with immobility often considered indicative of behavioral despair. In our study, AFIC-CDs demonstrated the ability to reduce immobility time, suggesting its potential as an antidepressant agent.

It is well known that the alterations in neurotransmitter function involving DA, 5-HT and NE may induce depression, and they are also the primary targets for currently available treatments of antidepressant drugs. 5-HT is a neuromodulator and neurotransmitter extensively distributed in the brain, which involved in the regulation of the basic physiological functions, such as development processes, energy expenditure and respiratory rate. 5-HT is related to a wide spectrum of human behavioral traits and neuropsychiatric disorders,a long-standing theory is that depression is caused by the deficiency of brain 5-HT and the selective 5-HT reuptake (SSRIs) are the first-choice antidepressant drug treatment in current time. The development of SSRIs further suggested that the depressive symptoms of depressed patients could be ameliorated by the increased 5-HT function. (Cowen, 2008; Gutknecht et al., 2012). DA regulates the reward system and mood, wise, decision-making and motivation. It has been shown that stress-induced DA release tightly coupled to the neuroendocrine stress responses. In executive functioning regulating cognition, intellect and motivation, NE plays a determinant role. The reduction of NE neurotransmission is related to decreased alertness, problems of concentration and low energy, and leads to the social dysfunction of depression patients, which maybe the most important factors affecting the life quality of patients. In this research, we evaluated the effect of AFIC-CDs on mouse brain cortex neurotransmitter levels, the results show the increase of 5-HT, DA and NE levels.

Neuroinflammation is one of the important points for the neuronal damage and the induction of various neurological diseases. Inflammation is an essential factor in the depression pathogenesis, the pro-inflammatory cytokines could intervene in neural processes such as neural plasticity and neural survival, which leads to the induction of depression (Wang et al., 2021b). Patients with major depression have been repeatedly observed to have activated inflammatory pathways, as manifested by increased proinflammatory cytokines such as TNF-α and IL-1β. The antidepressant strategies such as psychotherapy and medication appear to attenuate inflammatory activity and improve depression symptoms, which indicate that the inflammation reduction may be related to treatment of depression (Raison et al., 2006). In this research, we evaluated the effect of AFIC-CDs on mouse brain cortex cytokines levels, the results show the decrease of TNF-α and IL-1β levels, which indicate the anti-inflammatory activity of AFIC-CDs.

As a kind of critical regulators of the formation and plasticity of neuronal networks, BDNF levels can be a useful marker for clinical response or improvement of depressive symptoms. And promoting the upregulation of BDNF level has emerged as an effective way to ease depression. Patients with depression exhibited decreased atrophy and volumes of the hippocampus and prefrontal cortex, which indicate that neuronal damage may be one of the crucial mechanisms in the depression pathogenesis. In this research, we evaluated the effect of AFIC-CDs on mouse brain cortex BDNF level, the results show the increase in BDNF level, which indicate that the antidepressant activity of AFIC-CDs may be related to the increasing level of BDNF. Tph2 acts as rate-limiting enzymes in 5-HT synthesis (Gutknecht et al., 2008). The multiple polymorphisms of Tph2 have been associated with the response of antidepressant treatment (Siesser et al., 2013). In our research, we evaluated the effect of AFIC-CDs on Tph2 levels in the mouse brain cortex, and the results show an increase in Tph2 levels, which may be associated with the elevation of 5-HT levels.

## 4 Conclusion

In conclusion, bioactive natural ingredients may offer additional benefits in the treatment of depression compared to traditional single-target drugs, due to their low toxicity and improved efficacy. The current findings suggest that AFIC-CDs possess antidepressant activity, potentially acting by increasing the expression of BDNF and Tph2, elevating neurotransmitter levels such as 5-HT, DA, and NE, and reducing the levels of cytokines such as IL-1β and TNF-α. This research provides valuable insights for the development of novel antidepressant agents, positioning AFIC-CDs as a promising candidate for alleviating depression.

## Data Availability

The original contributions presented in the study are included in the article/Supplementary Material, further inquiries can be directed to the corresponding authors.
