# Supplementary figures and images for: Aurantii fructus immaturus carbonisata-derived carbon dots and their anti-depression effect
Source: Front Mol Biosci. 2024 Jan 8;10:1334083. doi: 10.3389/fmolb.2023.1334083 (PMC10801177; doi:10.3389/fmolb.2023.1334083)

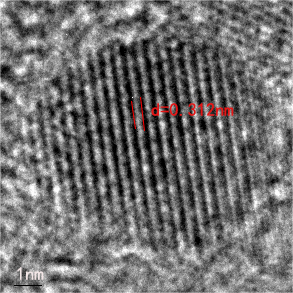

Supplement: Supplementary file 1 [file Image3.JPEG]

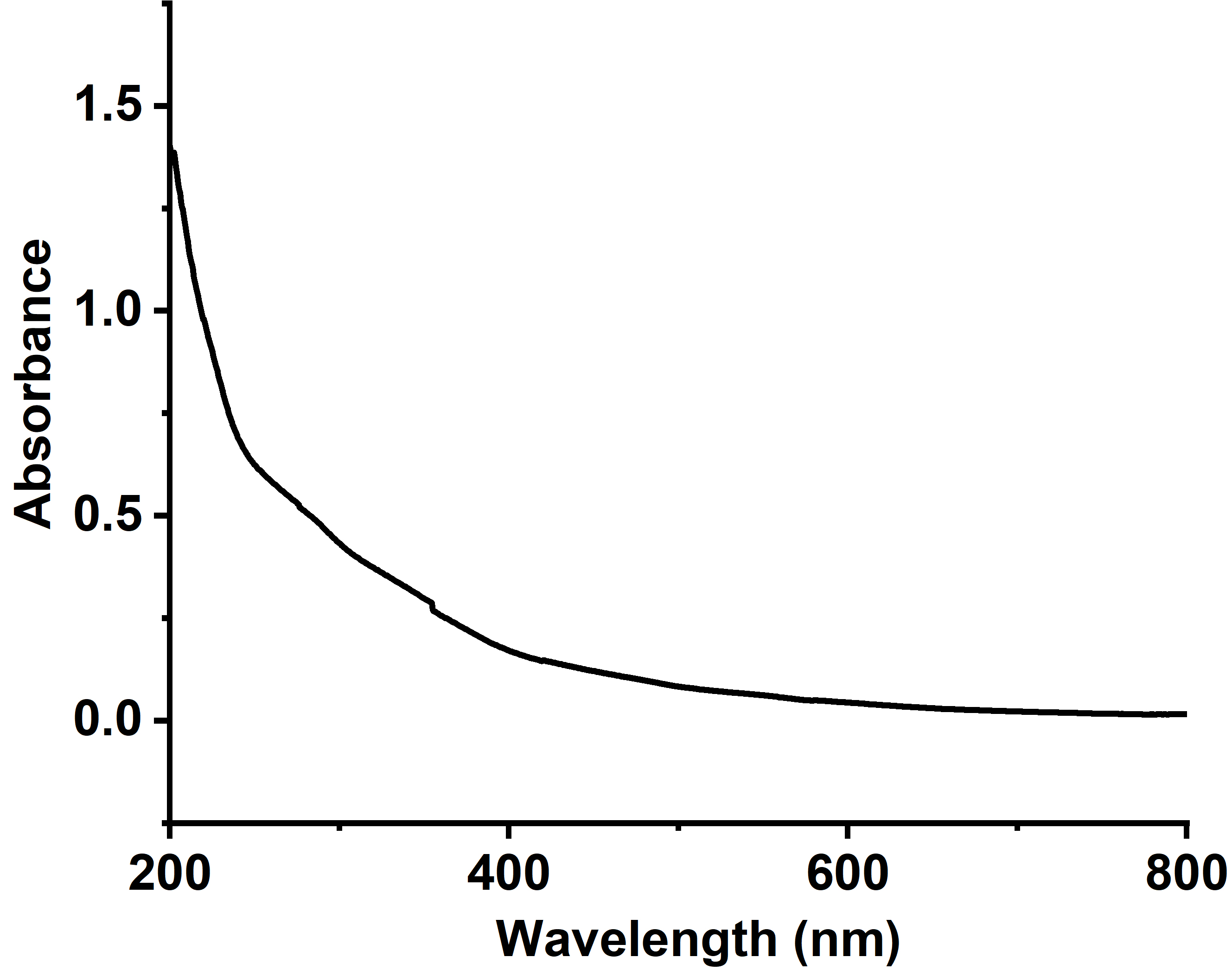

Supplement: Supplementary file 2 [file Image4.JPEG]

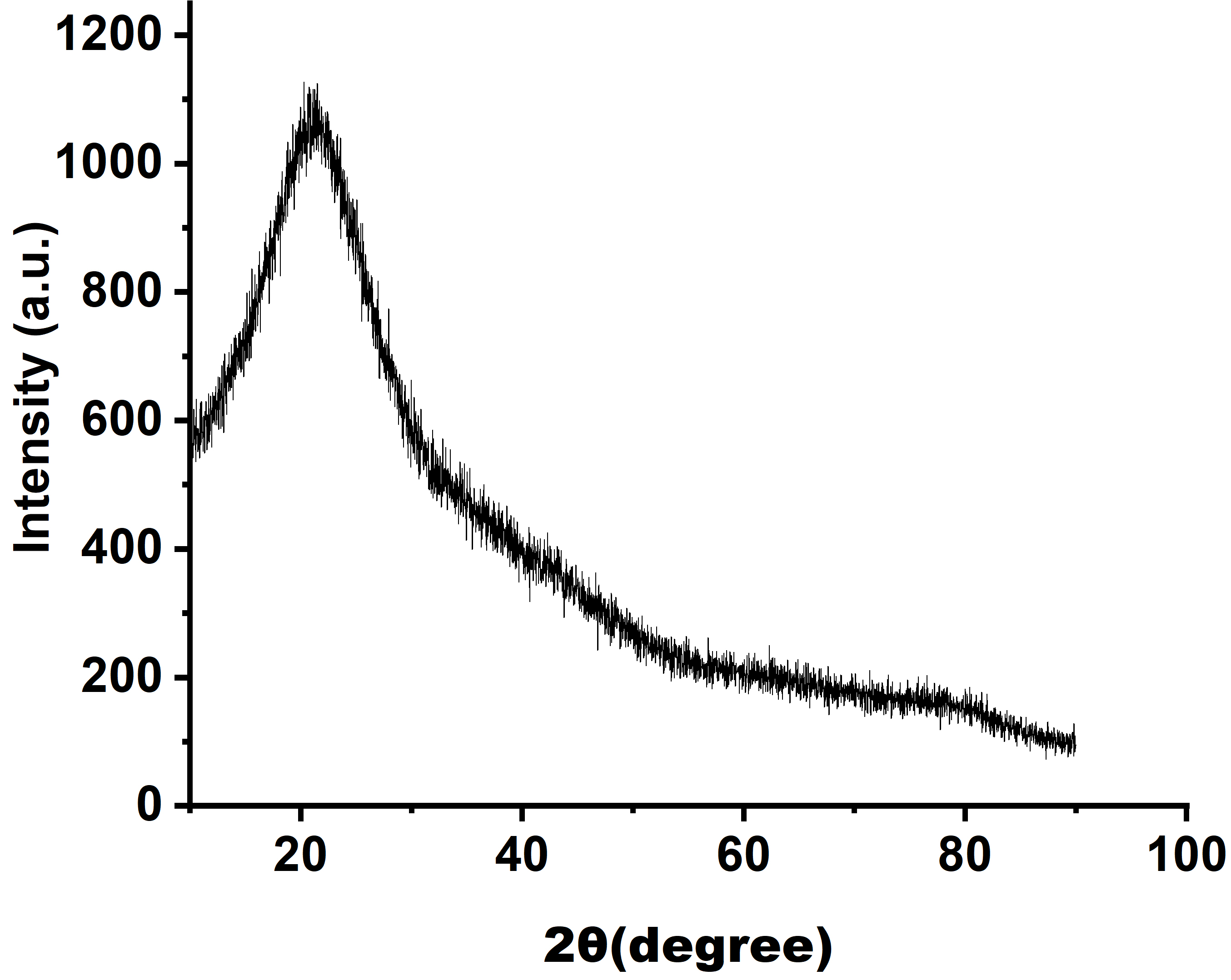

Supplement: Supplementary file 4 [file Image7.JPEG]

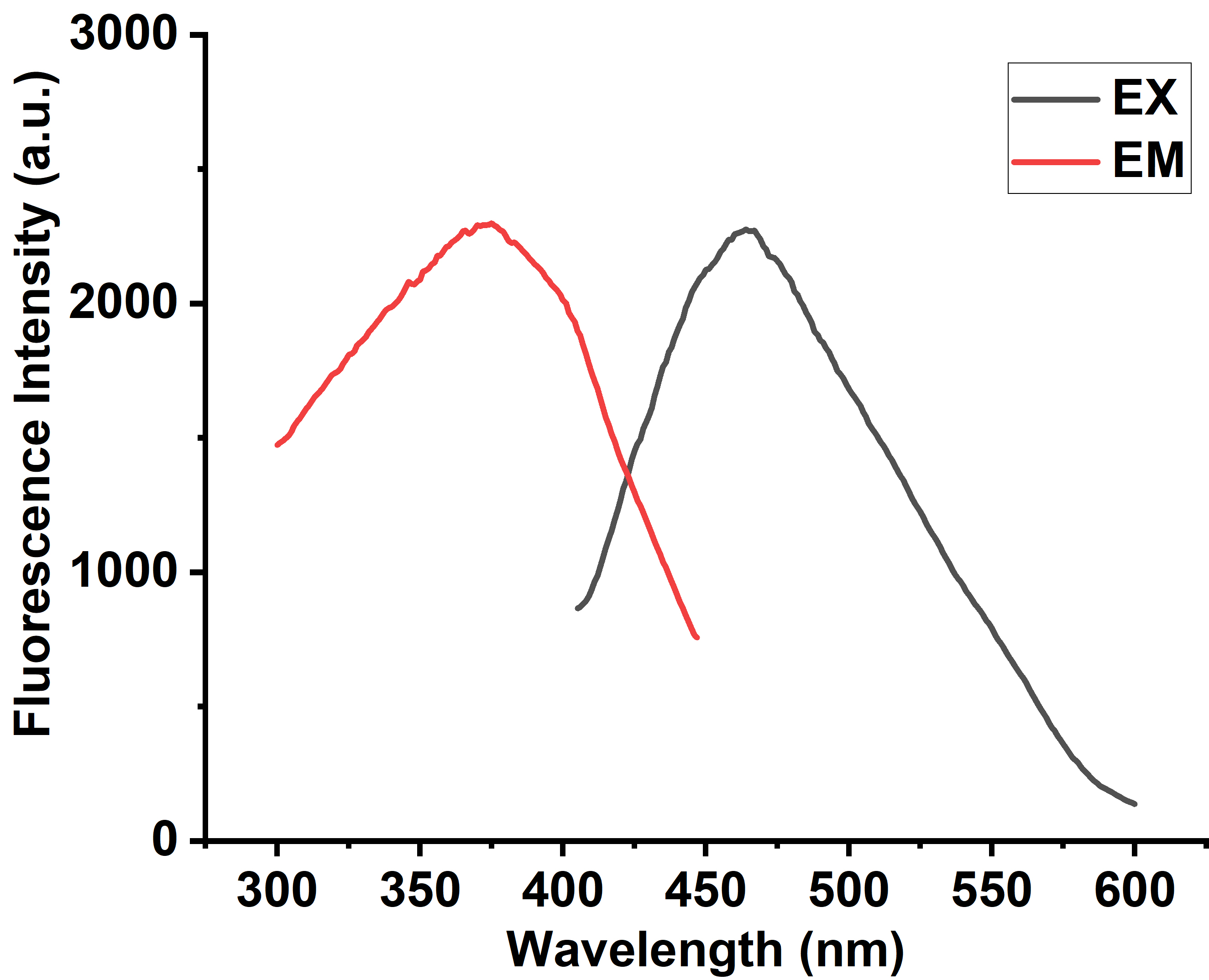

Supplement: Supplementary file 5 [file Image5.JPEG]

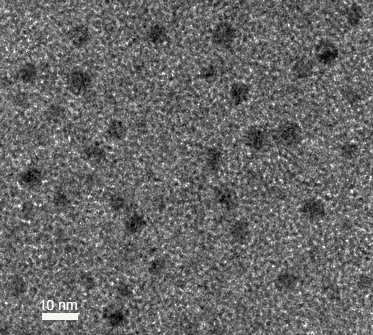

Supplement: Supplementary file 6 [file Image1.TIF]

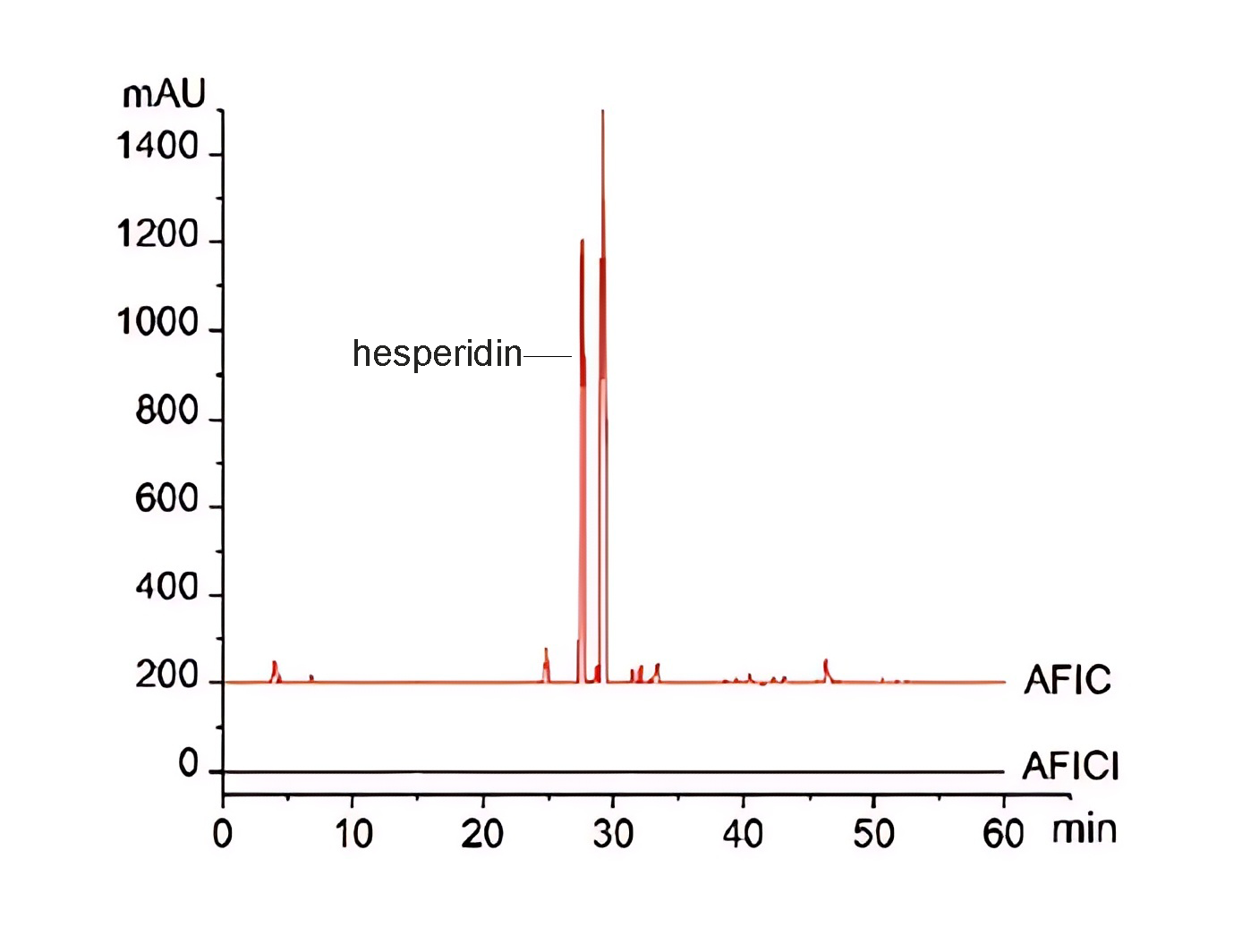

Supplement: Supplementary file 7 [file Image8.TIF]

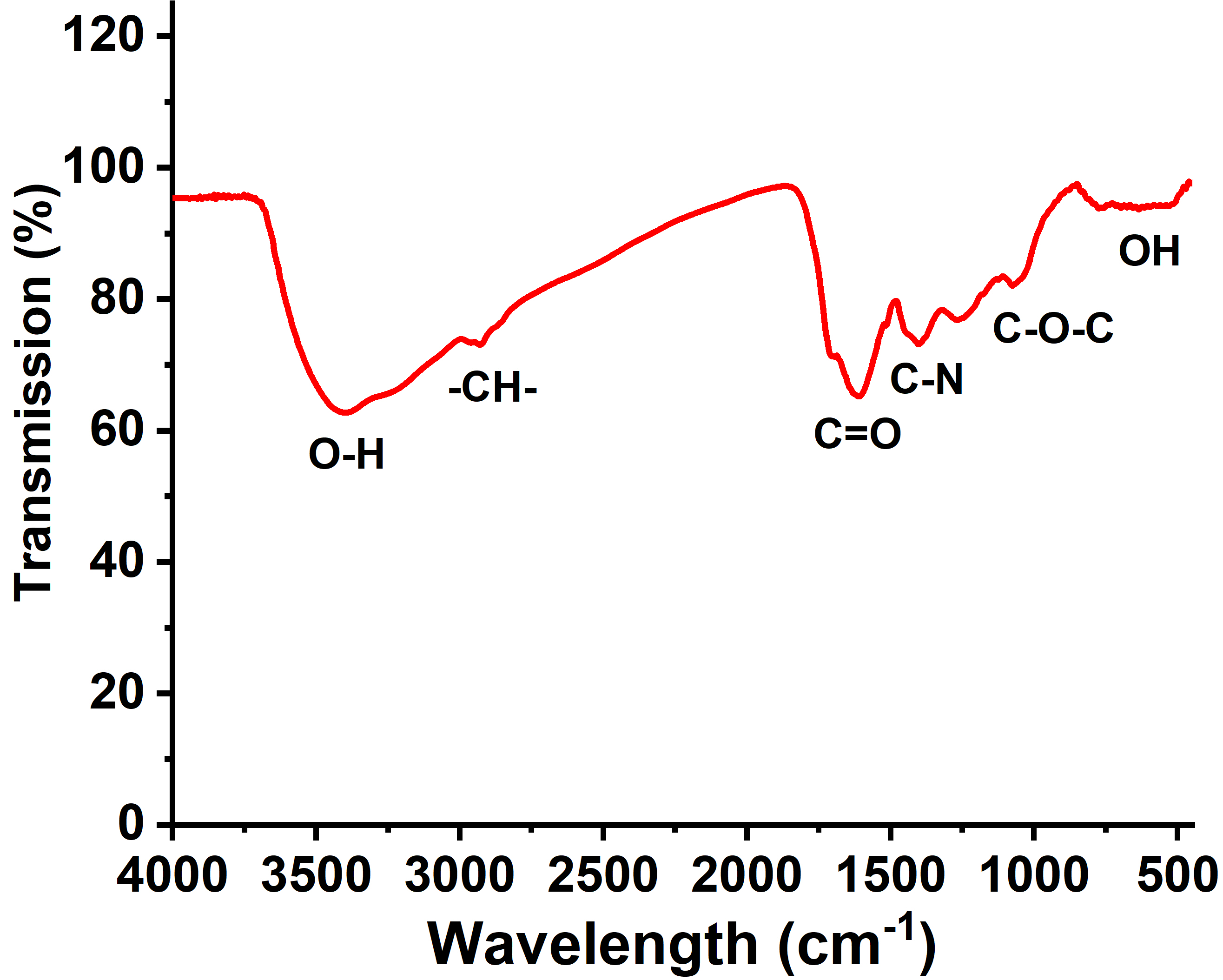

Supplement: Supplementary file 8 [file Image6.JPEG]
